# Elucidating the Molecular Mechanisms Underlying Fruit Bag‐Mediated Light Transmittance and Its Effects on Anthocyanin Biosynthesis in Chinese Plum *(Prunus salicina ‘Longzhong’)*: An Integrated Transcriptomic and Metabolomic Analysis

**DOI:** 10.1002/fsn3.72077

**Published:** 2026-07-15

**Authors:** Yanbiao Li, Jiangong Wang, Zhongxing Zhang, Yanlong Gao, Yang Liu, Qihua Lu, Jun Li

**Affiliations:** ^1^ College of Modern Agriculture, Jiaxing Vocational & Technical College Jiaxing Zhejiang China; ^2^ Key Laboratory of Characteristic Fruit and Vegetable Variety Improvement in Jiaxing Jiaxing China; ^3^ Jiaxing Academy of Agricultural Sciences Jiaxing China; ^4^ College of Horticulture Gansu Agricultural University Lanzhou China; ^5^ Horticultural Crop & Plum Research Institute in Jiaxing Jiaxing China

**Keywords:** *anthocyanin*, *bagging*, *metabolism*, *Prunus salicina*, *transcription*, *WGCNA*

## Abstract

Anthocyanins exert strong free radical scavenging effects to mitigate cellular oxidative damage, while enhancing fruit coloration and nutritional value. Here, *Prunus salicina ‘Longzhong’* (*Zuili plum*) was used as material. Four light transmittance bagging treatments (T1–T4) and a non‐bagged control (CK) were set, and combined transcriptome and metabolome analyses were performed to explore the molecular mechanism of light transmittance regulating anthocyanin biosynthesis. Anthocyanin accumulation was significantly positively correlated with light transmittance, and the total anthocyanin content under high transmittance (T1) was close to CK. High light induced the expression of key anthocyanin synthesis genes such as Chalcone Synthase (*CHS*), whereas low light upregulated upstream Phenylalanine Ammonia‐Lyase (*PAL*) but could not promote anthocyanin accumulation. Weighted Gene Co‐expression Network Analysis (WGCNA) identified core genes (*Asali03G034200*, *Asali04G026050*, *Asali01G050040*) closely correlated with anthocyanin content. Multi‐omics analysis confirmed the light transmittance‐gene‐metabolite regulatory pathway, with cyanidin‐3‐O‐glucoside serving as a light‐responsive marker. This study provides candidate genes for plum color improvement and a theoretical basis for precise bagging; future work will further explore bagging effects on overall fruit quality.

## Graphical abstract

*Zuili plum* fruits under CK and four bagging treatments (T1–T4) were analyzed via transcriptome and metabolome. The gradient fruit color phenotypes demonstrate that higher light transmittance facilitates anthocyanin accumulation in plum peel.

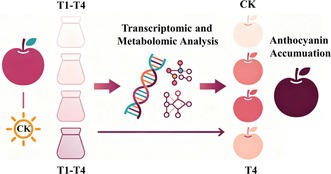

## 1 Introduction

Zuili (*Prunus salicina ‘Longzhong’*), a unique high‐quality plum resource endemic to China, is highly favored for its distinctive flavor and abundant nutritional value, boasting extremely high economic potential. Fruit external quality, particularly the intensity and uniformity of skin coloration, serves as a primary determinant of commercial value and consumer preference in the fresh fruit market. The core of this visual trait lies in the accumulation level and composition of anthocyanins in the fruit peel (Elikara et al. 2024). Anthocyanins are a class of water‐soluble flavonoid pigments that are widely distributed in plants (Habibi et al. 2025). Which not only determine the rich colors such as red, blue, and purple of fruits and flowers but also, as important antioxidants, are closely related to plant stress resistance and the health benefits of fruits (Wang et al. 2023).

The biosynthesis of anthocyanins follows a highly conserved metabolic pathway. Initiating from the phenylpropanoid pathway, it is catalyzed by key enzymes such as chalcone synthase (*CHS*), chalcone isomerase (*CHI*), and flavanone 3‐hydroxylase (*F3H*), and finally generates stable anthocyanins through the synergistic action of dihydroflavonol 4‐reductase (*DFR*), anthocyanidin synthase (*ANS*), and UDP‐glucosyltransferase (*UFGT*) (Hu et al. 2026; Muhammad et al. 2026). Among various environmental signals, light (including light intensity and light quality) is the most core factor regulating anthocyanin synthesis (Yang et al. 2026). Plants perceive light signals through photoreceptors such as phytochromes (Kondo et al. 2025), and cryptochromes (Pooja et al. 2014), and via a series of signal transduction processes (e.g., activation of the transcription factor HY5) (Xiao et al. 2025), they further crosstalk with the endogenous MBW regulatory network, ultimately synergistically inducing the expression of genes related to anthocyanin synthesis (Krylova et al. 2023). This indicates that anthocyanin synthesis is a precise outcome of the interaction between plant internal developmental programs and external light environments.

In fruit tree production practice, bagging technology is widely adopted to improve the external quality of fruits and reduce diseases and insect pests (Kumari et al. 2025). Its principle is to physically modify the microenvironment on the fruit surface, especially the light conditions (Georgoudaki et al. 2025). Although traditional bagging can effectively enhance fruit surface smoothness, it often causes coloration defects, which precisely confirms the indispensability of light in anthocyanin synthesis (Ding et al. 2025). Therefore, “fruit bag light transmittance”, as a core parameter of bagging technology, has become a key technical link affecting the final color performance of fruits (Mehdi et al. 2020). However, current studies on the effect of bagging on fruit coloration mostly focus on describing phenotypic phenomena, and the internal mechanism by which fruit bags with different light transmittances precisely regulate the “composition” and “content” of anthocyanins by modulating the molecular network of anthocyanin metabolism in *Zuili* fruits remains poorly understood.

To systematically address the aforementioned scientific issues, this study employed *Zuili plum* as the test material, established fruit bagging treatments with gradient light transmittance, and used non‐bagged fruits as the control. Samples were collected at two key fruit developmental stages (80 and 90 days after flowering), and a combination of transcriptomic and targeted anthocyanin metabolomic technologies was comprehensively applied. This study aims to clarify the molecular mechanisms by which the bagging microenvironment regulates fruit coloration in *Zuili*, providing valuable candidate genes for the genetic improvement of fruit color and a solid theoretical basis for formulating precise bagging cultivation strategies to optimize fruit external quality.

## 2 Materials and Methods

### 2.1 Plant Materials and Experimental Design

Seven adjacent rows of *Zuili* were used, with 60 plants per row. The two outermost rows were guard rows, and the middle 5 rows served as biological replicates for five treatments (CK, T1–T4), with 12 trees per treatment per replicate row. Routine field management was adopted. Fruits were bagged from 45 DAF to harvest (no bag removal), and fruits were cut with bags during sampling. Four types of wooden pulp bags (produced by Shandong Laitong Paper Co. Ltd.) were used: T1 (white, 50% light transmittance), T2 (striped, 15%), T3 (brown, 5%), T4 (double‐layer, 0%); non‐bagged fruits served as CK.

Sampling was conducted at 80 DAF (S1) and 90 DAF (S2). For each treatment, 15 plants were sampled (3 per replicate row), with 1 fruit per plant (upper/middle/lower canopy), totaling 45 fruits per treatment. Pericarp samples were snap‐frozen for transcriptome and anthocyanin detection; seed‐removed fruits for aromatic substance detection; and remaining fruits for quality determination. No repeated sampling was performed on the same plant.

Biological replicates: 5 field replicates (middle 5 rows). For omics analyses (transcriptomics, metabolomics), 3 independent replicates were set per treatment per sampling stage, with no sample pooling. Each replicate used tissues from a single independent plant. The experiment was conducted from April to July 2024. Sample labels: CK‐1/T1‐1‐T4‐1 (S2, July 7); CK‐2/T1‐2‐T4‐2 (S1, June 27).

### 2.2 Targeted Metabolomics Analysis

#### 2.2.1 Anthocyanin Extraction

After vacuum freeze‐drying, *Zuili plum* peel was ground into powder with a ball mill at 30 Hz for 1.5 min. Subsequently, 50 mg of the powder was weighed and homogenized in 500 μL of extraction solution (50% aqueous methanol containing 0.1% hydrochloric acid). The mixture was vortexed for 5 min, subjected to ultrasonic extraction for 5 min, and centrifuged at 12,000 rpm and 4°C for 3 min. The supernatant was collected, and the extraction procedure was repeated once. The two supernatants were pooled, filtered through a 0.22 μm microporous membrane, and transferred to sample vials for subsequent ultra‐high performance liquid chromatography–tandem mass spectrometry (UHPLC–MS/MS) analysis (Piryaei and Khiavi 2026).

#### 2.2.2 Detection Conditions

Sample analysis was performed using an ultra‐high performance liquid chromatography‐electrospray ionization‐tandem mass spectrometry (UPLC‐ESI‐MS/MS) system, consisting of an ExionLC AD UHPLC system (SCIEX) and an Applied Biosystems 6500 triple quadrupole mass spectrometer (SCIEX).

Chromatographic separation was performed on a Waters ACQUITY BEH C18 column (1.7 μm, 2.1 mm × 100 mm). Mobile phase A was ultrapure water supplemented with 0.5% formic acid, and mobile phase B was methanol with 0.5% formic acid. The gradient elution program was set as follows: 95:5 (A:B) at 0 min, linearly changed to 50:50 (A:B) at 6 min, adjusted to 5:95 (A:B) at 12 min and held for 2 min, then returned to 95:5 (A:B) at 14 min and equilibrated for 2 min. The flow rate was 0.35 mL/min, the column temperature was maintained at 40°C, and the injection volume was 2 μL (Daimon et al. 2019).

#### 2.2.3 Qualitative and Quantitative Methods

Standard curves were established using high‐purity anthocyanin standards. Standard solutions were prepared at a series of concentrations: 0.01 ng/mL, 0.05 ng/mL, 0.1 ng/mL, 0.5 ng/mL, 1 ng/mL, 5 ng/mL, 10 ng/mL, 50 ng/mL, 100 ng/mL, 500 ng/mL, 1000 ng/mL, 2000 ng/mL, and 5000 ng/mL. These solutions were analyzed under the same LC–MS/MS conditions as the samples. Chromatographic peak intensities corresponding to the quantitative signals of each standard at different concentrations were recorded. Standard curves for each compound were constructed with the concentration of the standard as the abscissa and the peak area as the ordinate. Subsequently, the peak area of each target anthocyanin in the test samples was substituted into the corresponding standard curve to calculate its exact content in the sample.

### 2.3 Data Preprocessing

Raw data files acquired by the LC–MS/MS instrument were directly uploaded to the Weikemeng Biotechnology Cloud platform. Metabolomic analysis in this study was performed using the high‐throughput targeted metabolomics workflow embedded on the platform. This workflow automatically completes core analytical procedures, including quality evaluation and quality control, normalization correction, basic statistical analysis, data visualization, and differential metabolite screening.

### 2.4 Transcriptomics Analysis

Sample RNA extraction, quality assessment, library construction, and high‐throughput sequencing will be performed by the Biomarker Technologies experimental team to ensure data quality. The raw sequencing data (Raw Data) generated will be in FASTQ format and uploaded directly to the client's personal project space on the Biomarker Cloud Platform.

Firstly, integrated data quality control tools will be utilized to evaluate the quality of the raw data, checking metrics such as sequencing error rate, GC content, and sequence length distribution. Subsequently, platform data filtering tools will be used to remove reads containing adapter sequences, low‐quality bases, and reads with an excessively high N content. This process yields high‐quality, clean sequencing data (Clean Data) for subsequent analysis. For transcriptome analysis, differentially expressed genes were identified with thresholds of adjusted *P* < 0.05 and |log_2_FC| > 1. The Benjamini‐Hochberg (BH) method was applied for multiple testing correction to control the false discovery rate (FDR).

For transcriptomic sequencing, 3 biological replicates were set for each treatment at each sampling time point. Specifically, after sampling, peel tissues from 3 randomly selected fruits (one fruit from each of 3 different trees) in each treatment were used as one biological replicate, and a total of 3 independent biological replicates were prepared per treatment per time point. No sample pooling was performed; each biological replicate was a separate sample derived from individual fruit peel tissue, ensuring the reliability and reproducibility of the transcriptomic data.

### 2.5 Targeted Metabolomic Analysis of Anthocyanins

LC–MS analysis was performed using an ultra‐high‐performance liquid chromatography coupled with Fourier transform mass spectrometry (UHPLC‐Q Exactive HF‐X) system.

Chromatographic conditions: An ACQUITY UPLC HSS T3 column (100 mm × 2.1 mm i.d., 1.8 μm; Waters, Milford, USA) was employed. Mobile phase A consisted of 95% water and 5% acetonitrile supplemented with 0.1% formic acid, while mobile phase B comprised 47.5% acetonitrile, 47.5% isopropanol, and 5% water with 0.1% formic acid. The injection volume was 2 μL, and the column temperature was maintained at 40°C. The gradient elution program was set as follows: 0–3.5 min, 100%–75.5% A; 3.5–5.0 min, 75.5%–35% A; 5.0–7.4 min, 100% B; 7.4–7.6 min, 48.5% A; 7.6–10.0 min, 100% A.

Mass spectrometry conditions: Electrospray ionization (ESI) was used for sample ionization, and mass spectral data were acquired in positive and negative ion scanning modes, respectively.

Raw data were imported into Progenesis QI metabolomics processing software (Waters Corporation, Milford, USA) for analysis, yielding a final data matrix containing information such as retention time, mass‐to‐charge ratio, and peak intensity. Principal component analysis (PCA) was conducted on the samples to evaluate the reliability of the predictive model. Differential metabolites were identified using screening criteria of *p* < 0.05 and variable importance in projection (VIP) > 1. KEGG pathway enrichment analysis was performed on the screened differential metabolites under different treatments to identify the color‐related differential metabolites of interest (Zhang et al. 2023). For metabolomic analysis (including anthocyanin and volatile compound determination), the same biological replication design as transcriptomics was adopted.

### 2.6 Weighted Gene Co‐Expression Network (WGCNA) Analysis

Differential genes and anthocyanin at different time points were used as trait files to generate co‐expression networks and modules. The Pearson correlation coefficient was calculated to determine the strength of the relationship between traits and modules. The co‐expression network was visualized using Cystoscope (version 3.6.0). The optimal soft threshold (power = 26) was determined to satisfy the scale‐free topology criterion. Genes were clustered into 22 modules based on TOM. Pearson correlation analysis was performed to correlate module eigengenes with anthocyanin content, and significant associations were identified at *p* < 0.01.

### 2.7 RT‐qPCR Analysis

Total RNA was extracted from samples using the SteadyPure Plant RNA Extraction Kit, and reverse transcription was subsequently performed with the Evo MLV RT‐qPCR Kit. The reverse‐transcribed cDNA was used as the template, with Actin selected as the reference gene. The qPCR reaction system was prepared as follows: 10 μL of 2 × SYBR Green Pro Taq HS, 1 μL of forward primer, 1 μL of reverse primer, 2 μL of cDNA template, and 6 μL of RNase‐free water. The amplification procedure was set as follows: initial denaturation at 95°C for 30 s, followed by 50 cycles of 95°C for 5 s and 60°C for 30 s. Three biological replicates were set for each sample. The detailed primer sequences are listed in Table S1.

### 2.8 Statistical Analysis

All experiments were conducted with at least 3 biological replicates. One‐way ANOVA was performed using SPSS 25.0 software, and Duncan's New Multiple Range Test was used for multiple comparisons between groups. A *p* < 0.05 was considered as the criterion for statistically significant differences.

## 3 Results

### 3.1 Effects of Fruit Bags With Different Light Transmittances on Anthocyanin Accumulation

**FIGURE 1:**
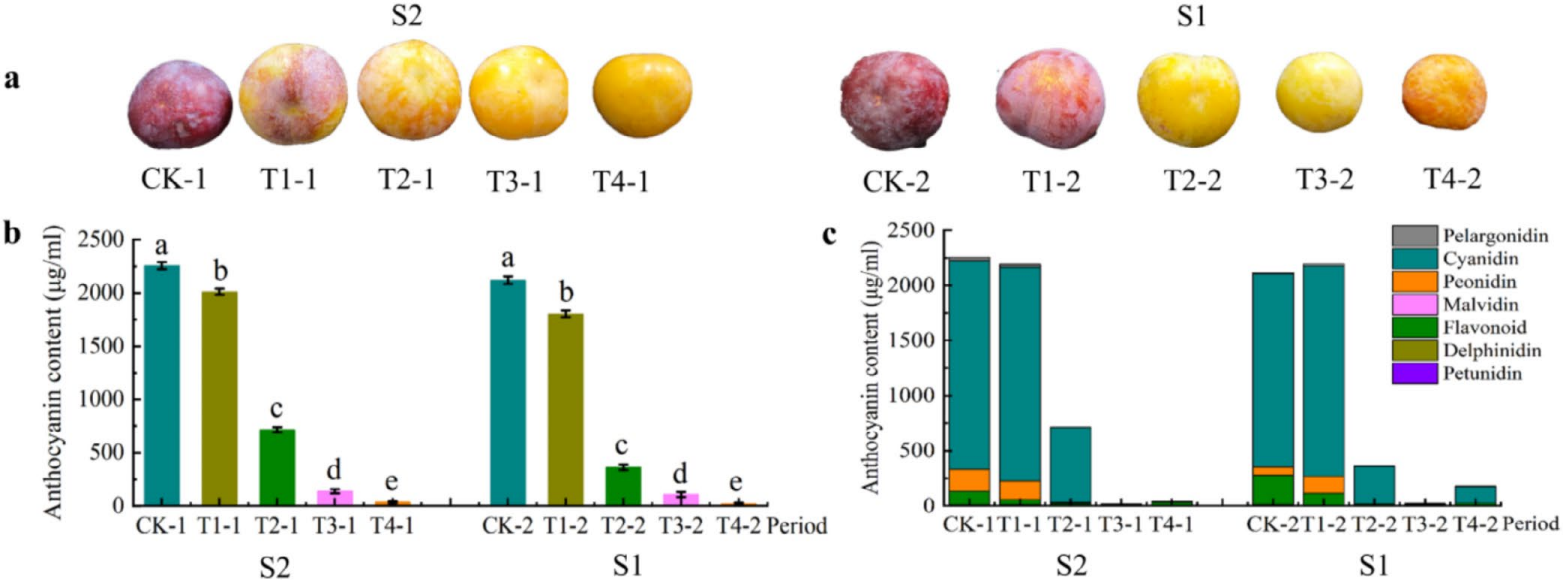
The effects of different treatments on the anthocyanin content of fruits. (a) Fruit coloration phenotypes of *P. salicina* at two developmental stages under different gradient treatments. (b) Anthocyanin content, (c) Content of major anthocyanin monomers. Note: Different lowercase letters represent significant differences between different treatments.

The fruit peel color of *P. salicina* varied markedly across different bagging treatments at two developmental stages (S1 and S2) (Figure 1a). At the S1 stage, control fruits (CK‐1) displayed a deep purplish‐red phenotype, whereas fruit color intensity gradually decreased from T1 to T4. A similar color variation tendency was observed at the S2 stage; CK‐2 fruits presented the darkest purplish‐red color, and peel color continuously faded among treatments from T1 to T4.

Fruit anthocyanin accumulation was significantly altered by different bagging regimes (Figure 1b). The CK group possessed the highest anthocyanin concentration, with values of 2255.08 μg/mL and 2118.70 μg/mL at the S1 and S2 stages, respectively, demonstrating that high light transmittance efficiently facilitates anthocyanin biosynthesis in *Zuili plum*. Fruit under the T1 treatment also exhibited high anthocyanin contents, measuring 2189.76 μg/mL and 2102.38 μg/mL at stages S1 and S2, respectively. In contrast, treatments with lower light transmittance (T2, T3, T4) showed a decreasing trend in anthocyanin content as transmittance diminished. Specifically, under the T4 treatment, the anthocyanin content at stage S2 was only 20.88 μg/mL, which was significantly lower than that of other treatments.

In terms of composition, cyanidin and pelargonidin were the major anthocyanin components, with cyanidin accumulating more significantly under high light transmittance conditions. Total anthocyanin content increased in all treatments as the fruit developed and ripened from stage S1 to S2, with the most pronounced increases observed in the CK and T1 treatments (Figure 1c).

**FIGURE 2:**
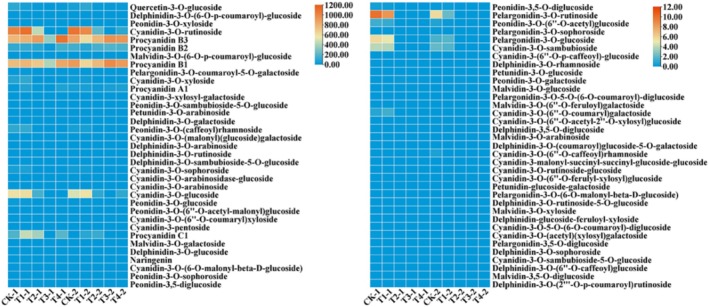
Specific content of anthocyanins in fruits under different treatments.

Qualitative and quantitative analysis of anthocyanins revealed that fruit bagging treatments with different light transmittances significantly influenced both the composition and content of anthocyanins in *Zuili plum* peel (Figure 2). In the high light transmittance groups (CK and T1), the contents of various anthocyanins were significantly upregulated. Major compounds included Cyanidin‐3‐O‐rutinoside, Cyanidin‐3‐O‐glucoside, Pelargonidin‐3‐O‐rutinoside, and Pelargonidin‐3‐O‐glucoside. Specifically, the content of Cyanidin‐3‐O‐rutinoside in the CK group reached 1200.00 μg/mL, which was significantly higher than in other treatments. The anthocyanin accumulation pattern in the T1 group was similar to that of the CK group, though overall levels were slightly lower. As light transmittance decreased (T2, T3, T4), the contents of most anthocyanins exhibited a clear downward trend. In the T4 treatment, which had the lowest light transmittance, the contents of all detected anthocyanins were close to 0 μg/mL. Regarding composition, derivatives of Cyanidin, Peonidin, and Delphinidin were the primary accumulated types. High light conditions were more conducive to the synthesis and accumulation of these substances (Table S2). These results indicate that light is a key factor regulating anthocyanin synthesis in *Zuili plum* peel, and reduced light transmittance significantly inhibits anthocyanin biosynthesis and accumulation.

### 3.2 Transcriptomic Analysis of *Zuili plum* Fruit Under Different Treatments

We performed systematic quality control on the raw sequencing data of all samples. The results showed that each sample generated more than 5.9 Gb of valid data with adequate clean reads, and the sequencing depth fully satisfied the requirements for transcriptome analysis. The percentage of unknown bases (*N*%) was 0 in all samples, indicating no base deletion. The GC content was stably distributed between 44.90% and 46.05%, with no exogenous nucleic acid contamination detected. In terms of base quality, the Q20 and Q30 values of all samples were above 99.47% and 97.07%, respectively. All quality indicators were considerably higher than the general criteria for transcriptome sequencing, which confirms the high quality of our sequencing data and ensures its availability for subsequent analyses (Table S3).

**FIGURE 3:**
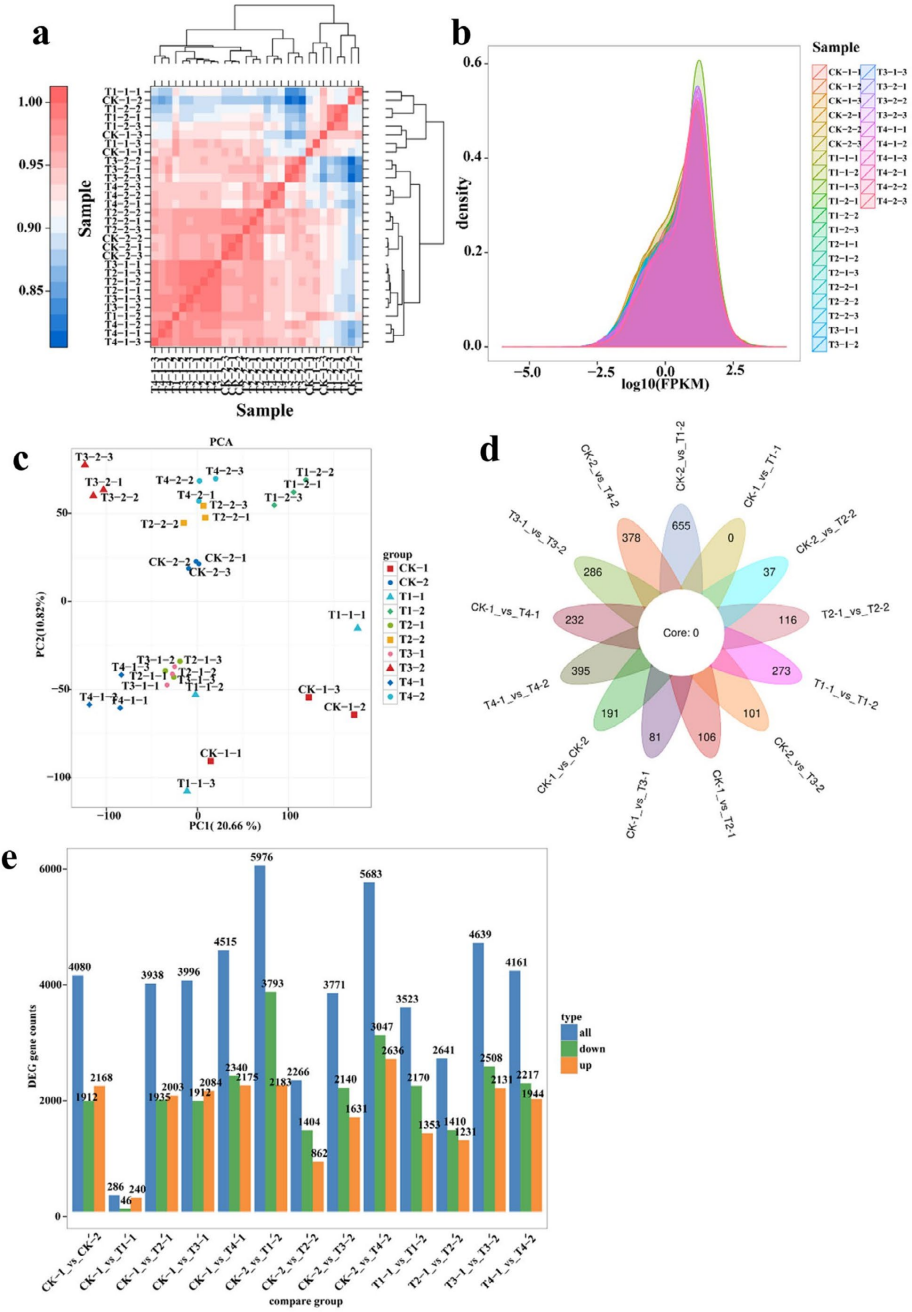
Correlation analysis among transcriptome samples (a), gene expression distribution kernel density curve (b), principal component analysis (c), gene Venn diagram (d), and statistics of differentially expressed genes (e).

Sample correlation analysis revealed obvious sample clustering across different treatments. CK‐1 and CK‐2 clustered tightly, indicating a high correlation coefficient (red region). With the decrease in light transmittance, samples of T1, T2, and T3 formed independent branches. Notably, T4 samples showed the most distinct expression profile and the lowest correlation with other groups (blue region). Overall, the clustering results indicated that changes in light transmittance markedly reshaped the gene expression patterns of plum fruits (Figure 3a).

Gene expression distribution exhibited obvious differences among experimental groups (Figure 3b). The relatively wider curves obtained from the CK and T1 groups indicated a broader expression range for both highly and lowly expressed genes. The curves for CK and T1 were relatively wider, indicating a broader range of both highly and lowly expressed genes. Conversely, as light transmittance decreased, the density curves for T3 and T4 peaked at moderate expression levels (log10 (FPKM) ≈1–2); the abbreviation FPKM stands for Fragments Per Kilobase of transcript per Million mapped reads, which represents a commonly used index for gene expression quantification. This suggests that shading conditions constrained gene expression to lower and medium levels, likely reflecting a global regulatory effect of attenuated light signals.

PCA demonstrated clear separation of treatments in the two‐dimensional space defined by PC1 (20.66%) and PC2 (10.82%) (Figure 3c). CK and T1 samples clustered together, T2 and T3 were in adjacent regions, and T4 formed a completely distinct cluster. The significant separation along the PC1 axis indicates that light conditions accounted for the primary variation in gene expression. This pattern not only confirms the transcriptional divergence caused by different light transmittances but also validates the significant impact of developmental stages (S1 vs. S2) on sample clustering within the same treatment.

Differentially expressed genes (DEGs) across distinct comparison groups exhibited no overlapping profiles (Figure 3d). The central label “Core: 0” confirmed that no DEGs were shared across all groups. Each discrete colored region corresponds to one of the eight comparison groups (e.g., CK‐2 vs. T1‐1, T1‐2 vs. T1‐1) and indicates the respective number of DEGs in each group. These results demonstrate that the combined effects of bagging treatment and fruit developmental stage triggered highly specific transcriptional responses. Notably, no universal core regulatory gene set was found to respond consistently to all experimental conditions.

Regarding the number of DEGs (Figure 3e), comparisons between different developmental stages under the same treatment (e.g., T1‐2vsT1‐1) generally yielded more DEGs than comparisons between different treatments at the same stage (e.g., CK‐2vsT1‐1). Furthermore, down‐regulated genes outnumbered up‐regulated genes in all comparisons. Notably, the T4 treatment exhibited the most dramatic changes in DEG counts between stages, further confirming the profound influence of the light environment on fruit gene expression.

Functional classification based on Clusters of Orthologous Groups (COG) revealed a marked difference in the distribution of consensus sequences across functional categories under different treatments. Among all categories, Category Q (biosynthesis, transport, and catabolism of secondary metabolites) exhibited the highest abundance and represented the most enriched functional class. This finding suggests that the strain possesses a prominent capability for secondary metabolite biosynthesis and transport, which may be closely associated with its environmental adaptability and competitive strategy.

The second and third most abundant categories were Category O (translation, protein turnover, and molecular chaperones) and Category C (energy production and conversion), implying that energy metabolism and protein homeostasis play essential roles in the physiological activities of this strain. Additionally, Category J (translation, ribosomal structure, and biogenesis) and Category R (general function prediction only) showed relatively high frequencies, indicating that fundamental protein synthesis and general functional genes underpin its core physiological processes. In comparison, functional categories A, D, F, H, L, N, P, U, W, X, Y, and Z all displayed a gene frequency of zero, suggesting that these functions are either entirely absent or not encoded in the genome of this strain (Figure S1a).

From the GO functional classification bar chart of this transcriptome, it can be seen that the distribution of genes in the three major categories of cellular components, molecular functions, and biological processes shows a clear preference. Among the cellular groups, the cellular anatomical entities account for the largest proportion, indicating that the internal structures and complex assembly within the cells are the core cellular characteristics of this sample. In terms of molecular functions, catalytic activity and binding are the most prominent. Among them, antioxidant activity and molecular transduction activity also account for a certain proportion. This indicates that the genes in this sample are mainly involved in enzymatic reactions and molecular interaction processes. In biological processes, the enrichment levels of metabolic process and cellular process are the highest. Processes such as reproduction and immune system also show significant expression, demonstrating the dominant role of basic metabolism and cellular life activities (Figure S1).

**FIGURE 4:**
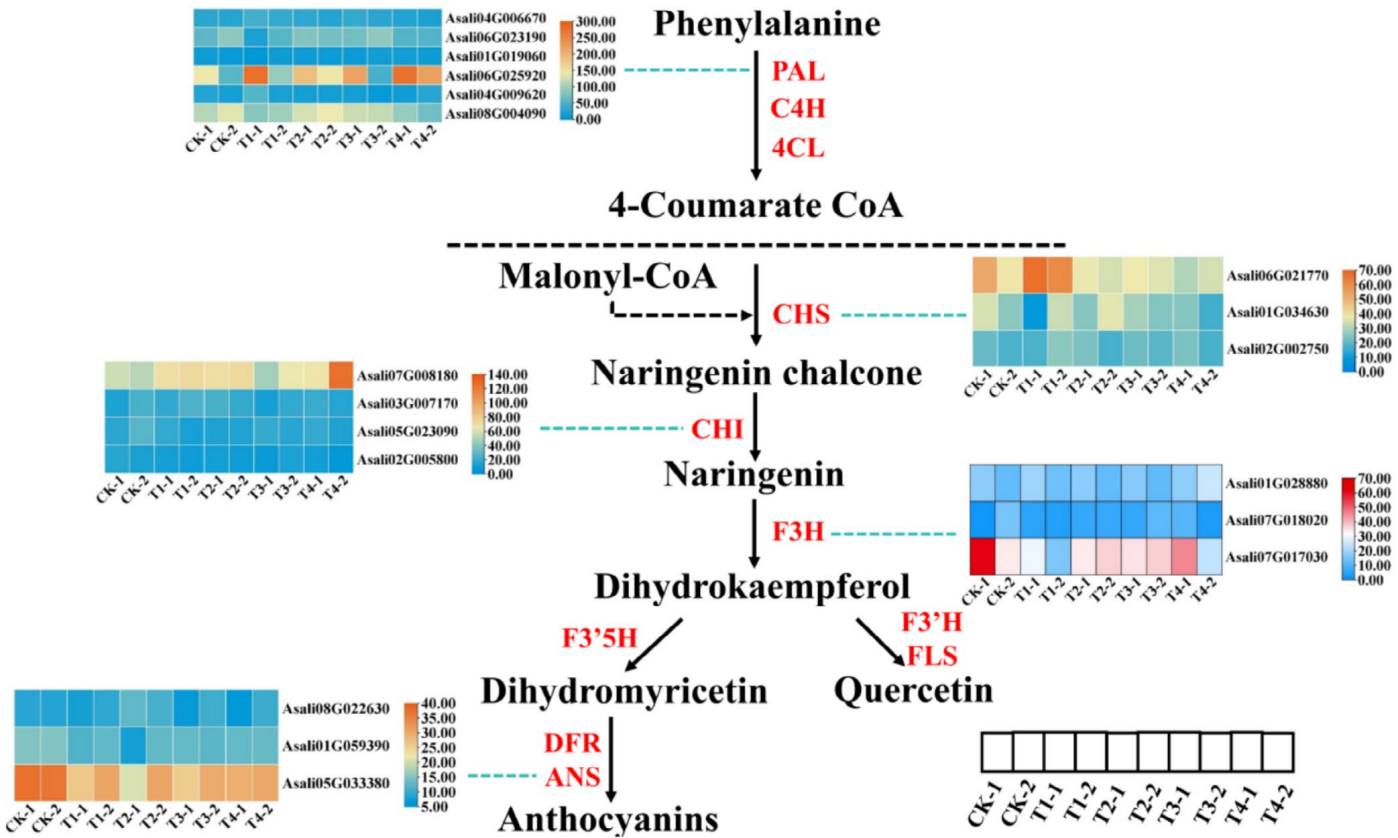
Anthocyanin biosynthesis pathway. The color gradient represents gene expression levels normalized by FPKM.

As shown in Figure 4. The genes encoding phenylalanine ammonia‐lyase (*PAL*), cinnamate 4‐hydroxylase (*C4H*), and 4‐coumarate‐CoA ligase (*4CL*) were significantly upregulated in the T3‐2 and T4‐2 treatment groups, displaying a clear time‐dependent induction pattern. This provides sufficient 4‐coumaroyl‐CoA precursors for downstream metabolism. Chalcone synthase (*CHS*) and chalcone isomerase (*CHI*) are key enzymes in flavonoid biosynthesis. *CHS* family genes exhibited higher expression levels in the T1 and T2 stages, whereas *CHI* genes maintained basal expression across all treatments.

At the metabolic branch point, flavanone 3‐hydroxylase (*F3H*) genes were specifically highly expressed in CK‐1 and CK‐2, while flavonol synthase (*FLS*) genes showed no significant changes in expression across all treatments, indicating that the experimental treatments primarily promoted the anthocyanin biosynthetic branch rather than the flavonol branch. Genes encoding dihydromyricetin synthase (*F3′5’H*), dihydroflavonol 4‐reductase (*DFR*), and anthocyanidin synthase (ANS)—key enzymes in anthocyanin synthesis—were significantly upregulated at the T4‐2 stage, which was highly consistent with the temporal trend of anthocyanin accumulation, thus revealing the transcriptional regulatory mode of this pathway.

### 3.3 Metabolomic Analysis of Anthocyanins in *Zuili plum* Fruit Under Different Treatments

In terms of metabolite distribution, acylated anthocyanin derivatives (e.g., caffeoyl and coumaroyl modifications) were more sensitive to light conditions, whereas simple glycosylated anthocyanins showed relatively moderate changes. This indicates that light not only affects total anthocyanin accumulation but also modulates the composition and chemical structure of anthocyanins by regulating acylation and glycosylation, providing important metabolomic evidence for improving fruit color quality via light management.

**FIGURE 5:**
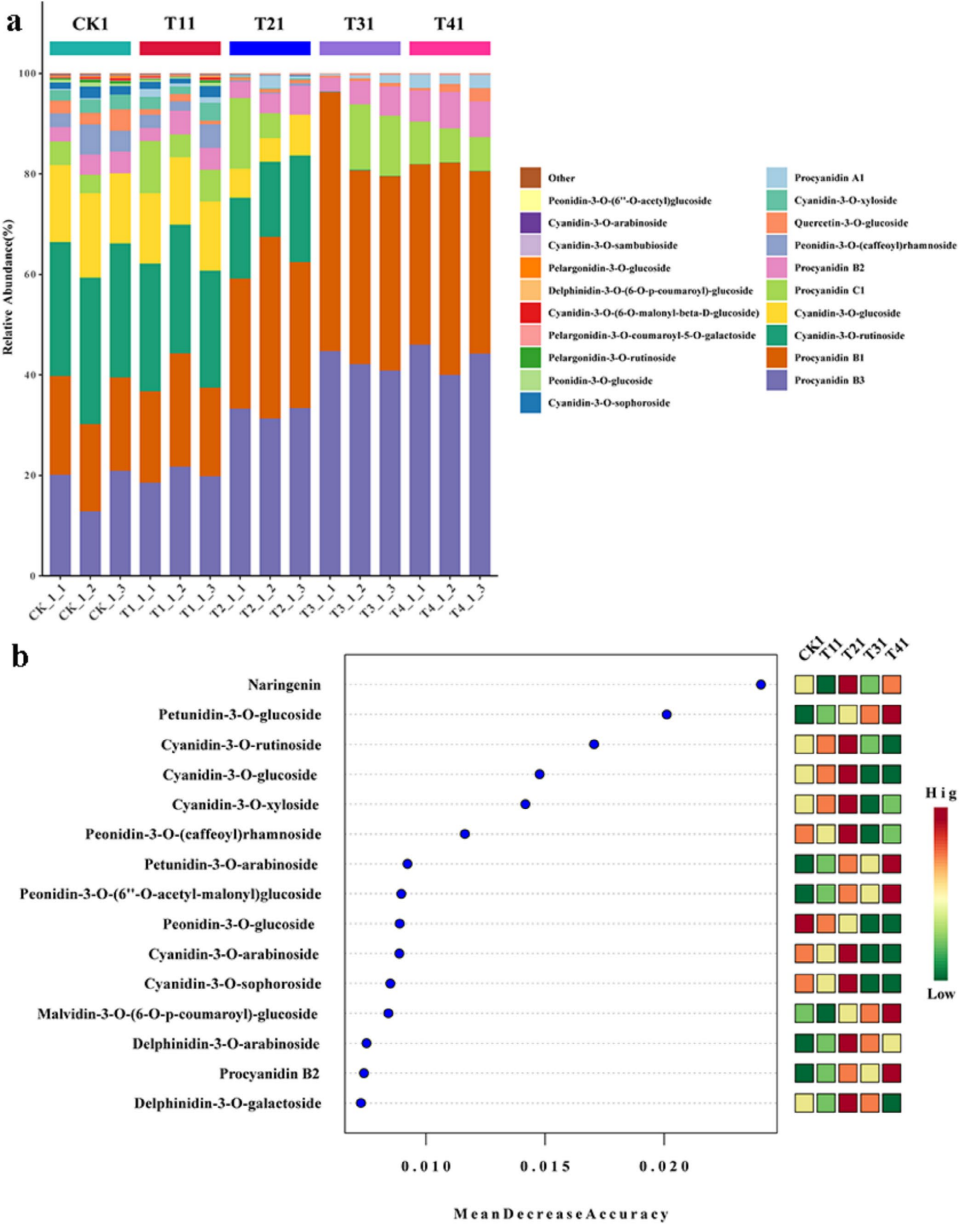
All group‐clustered Pearson correlation heatmap (a) and variable importance analysis (b).

Anthocyanins and flavonoids, the main secondary metabolites, accumulated to significantly higher levels in CK and T1 compared with low‐light treatments (Figure 5a). Major anthocyanins such as Cyanidin‐3‐O‐glucoside and Pelargonidin‐3‐O‐glucoside reached their highest levels in CK, decreased gradually with lower light transmittance, and were lowest in T4. Meanwhile, intermediate and primary metabolites showed smaller changes, suggesting that light primarily affects specific secondary metabolic pathways rather than global metabolism.

Scatter plot analysis screened key differential metabolites based on variable importance in projection (VIP) values, with the highest contribution to the classification model. As shown in Figure 5b, the data points on the right correspond to metabolites with remarkable intergroup differences and strong discriminatory capacity, mainly comprising various anthocyanins and their derivatives. These metabolites had VIP values greater than 1.0 and elevated −log_10_(FDR) values, indicating extremely significant differences among treatments and enabling them to serve as key biomarkers for distinguishing different light conditions. Notably, the points at the far right with the highest VIP values likely represent the core metabolites most intensely regulated by light.

**FIGURE 6:**
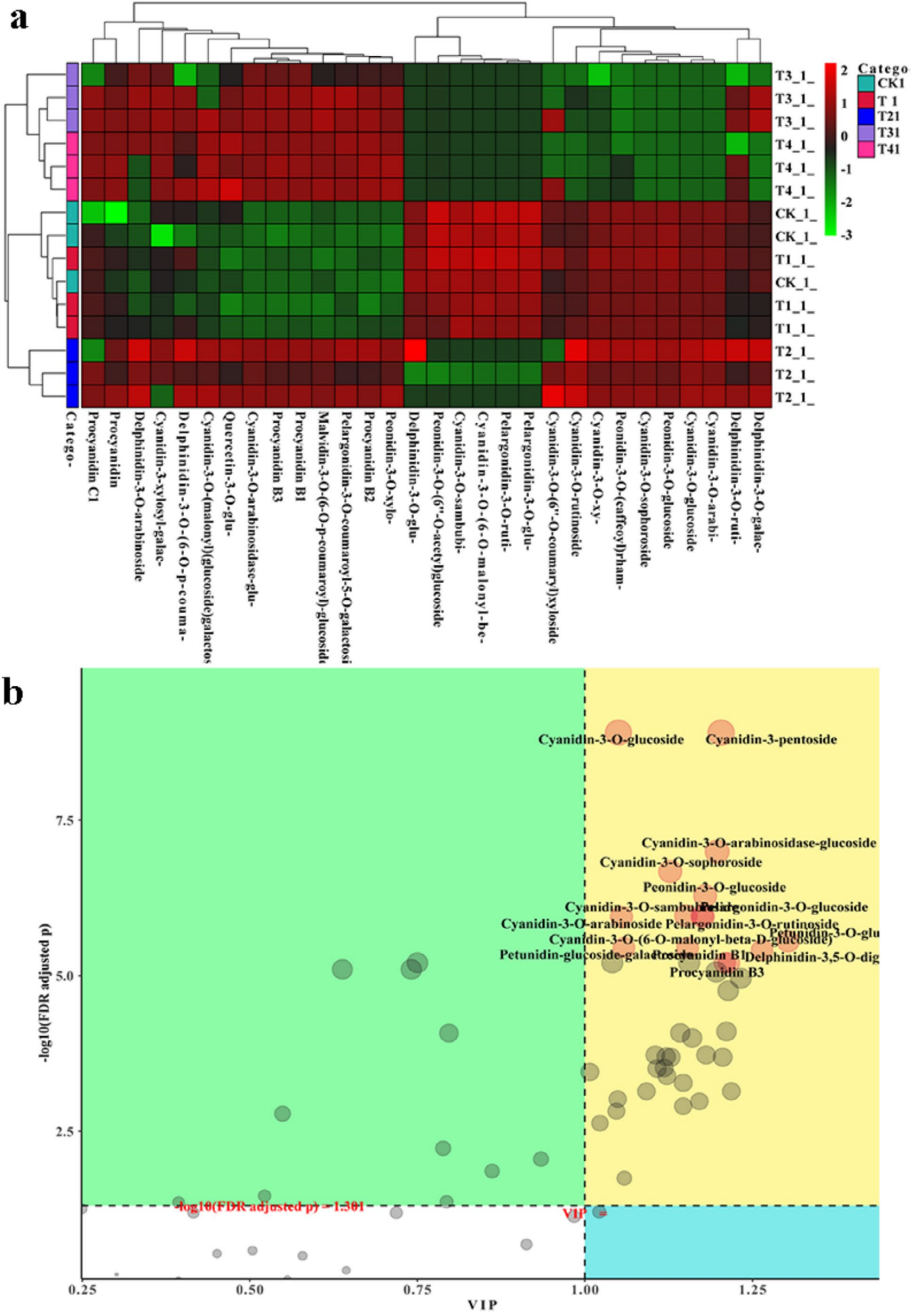
Metabolite expression heatmap (a) and scatter plot of significantly different metabolites (b).

The metabolite expression heatmap (Figure 6a) revealed distinct abundance patterns of major anthocyanins, including cyanidin‐3‐O‐glucoside, cyanidin‐3‐O‐galactoside, and pelargonidin‐3‐O‐glucoside, across different treatments. Overall, anthocyanin relative abundances were markedly higher in the CK and T1 groups. As light transmittance decreased with declining bagging permeability (T2 and T3), anthocyanin levels declined gradually, reaching the minimum under the T4 treatment. These findings indicate that light intensity acts as a crucial environmental factor modulating the accumulation of key pigment‐related anthocyanin metabolites.

In Figure 6b, metabolites with VIP > 1.0 and elevated −log_10_(FDR) values possessed statistical significance and strong contributions to the classification model, encompassing multiple annotated anthocyanins. These compounds are potential key quality biomarkers that respond sensitively to varying light environments. Their abundance patterns were highly consistent with the heatmap results, further confirming that light directly modulates fruit appearance quality and internal nutritional properties by substantially regulating the accumulation of secondary metabolites such as anthocyanins in *P. salicina* fruit.

### 3.4 Integrated Analysis of Transcriptomics and Metabolomics

#### 3.4.1 Screening of Soft Threshold and Verification of Scale‐Free Network

To construct a gene co‐expression network conforming to scale‐free topological characteristics, this study systematically screened and evaluated the optimal value of the soft threshold (power). As shown in Figure S2a, the signed R^2^ of the scale‐free topology fitting model exhibited a continuous upward trend with the increase of the power value. When the power was set to 26, the signed R^2^ reached 0.81, satisfying the core criterion (R^2^ ≥ 0.8) required for scale‐free networks in WGCNA analysis. Meanwhile, the Figure 8a demonstrated that the mean connectivity of the network remained at a reasonable level under power = 26, which not only ensured the moderate sparsity of the network but also retained sufficient inter‐gene association information. Therefore, power = 26 was ultimately selected as the optimal soft threshold for the subsequent construction of the co‐expression network.

Based on the selected optimal soft threshold (power = 26), the scale‐free topological characteristics of the network were further verified. The connectivity histogram in Figure 8b showed a typical right‐skewed distribution of gene connectivity, indicating that the vast majority of genes had low connectivity, while only a small number of genes were high‐connectivity hub genes, which is consistent with the core characteristics of scale‐free networks. The log_10_(p (k))–log_10_(ck) fitting plot in Figure S2b further confirmed that the scale‐free fitting R^2^ of the network was 0.81, with a fitting slope of −1.24, which was highly close to the theoretical slope (−1) of scale‐free networks. These results indicated that the constructed co‐expression network strictly satisfied the scale‐free topology assumption, with a stable and reliable network structure, and could be used for subsequent gene module division and module‐trait correlation analysis.

#### 3.4.2 Gene Module Partition and Module‐Anthocyanin Trait Correlation

**FIGURE 7:**
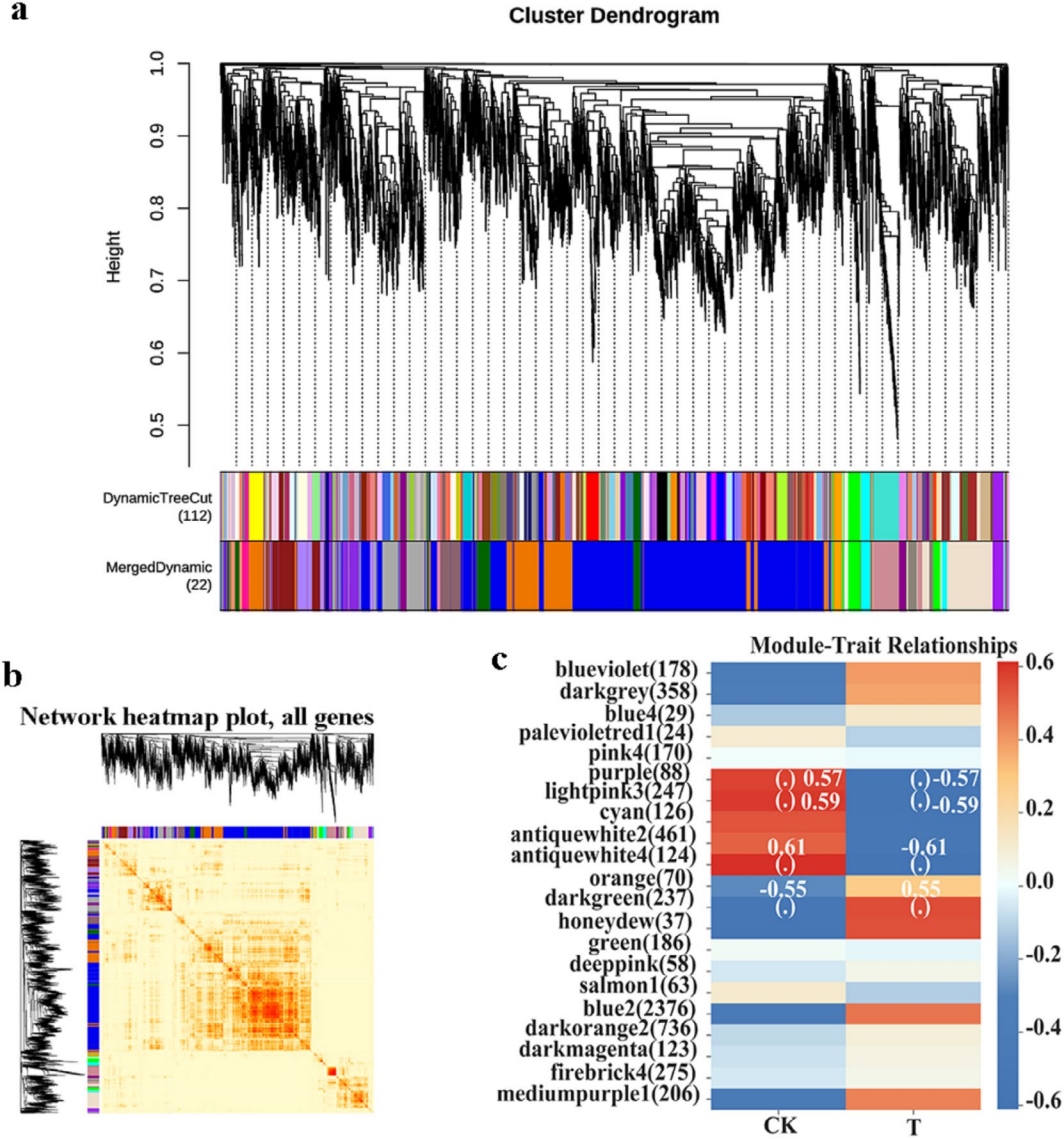
Analysis on the construction of gene co‐expression modules and module‐trait associations in WGCNA. (a) Cluster dendrogram. (b) Network heatmap plot, all genes. (c) Module‐trait relationships; both columns of indicators represent the total anthocyanin content.

Based on the optimal soft threshold (power = 26) identified previously, gene co‐expression networks were constructed and modules were partitioned via WGCNA. As shown in Figure 7a. Hierarchical clustering was performed on all expressed genes, and 112 initial gene modules were obtained using the DynamicTreeCut method. Subsequently, modules with high similarity were merged, resulting in 22 non‐gray co‐expression modules. The gene expression patterns within each module were highly consistent, while the expression profiles differed significantly among distinct modules, indicating a stable and reliable module partitioning result.

The reliability of the network structure was further verified by the gene co‐expression heatmap in Figure 7b. In the heatmap, orange/red regions represent high co‐expression correlations between genes, and blue regions indicate low correlations. It is evident that genes within the same module form distinct high‐coexpression clusters, whereas the co‐expression correlations between different modules are substantially reduced. This pattern directly validates the effectiveness of module division and confirms that the co‐expression network exhibits a typical scale‐free topological structure.

To identify key gene modules significantly associated with anthocyanin content (the target trait), the Pearson correlation coefficients between each module and anthocyanin content were calculated. As illustrated in Figure 7c. Multiple modules showed strong correlations with anthocyanin content. Specifically, the purple module (*r* = 0.57, *p* < 0.01), lightpink3 module (*r* = 0.50, *p* < 0.01), cyan module (r = 0.61, *p* < 0.01), and antiquewhite2 module (*r* = 0.61, *p* < 0.01) exhibited extremely significant positive correlations with anthocyanin content. In contrast, the darkgreen module showed an extremely significant negative correlation (r = −0.55, *p* < 0.01) with anthocyanin content. These findings suggest that the positively correlated modules are enriched with key genes that positively regulate anthocyanin biosynthesis, while the negatively correlated module likely harbors negative regulators of anthocyanin synthesis. This not only provides crucial evidence for screening core candidate genes related to fruit coloration but also lays a foundation for elucidating the molecular regulatory network of anthocyanin accumulation, thereby achieving effective cross‐validation between transcriptomic and metabolomic data.

### 3.5 Co‐Expression Correlation Analysis of Transcriptome and Metabolome

**FIGURE 8:**
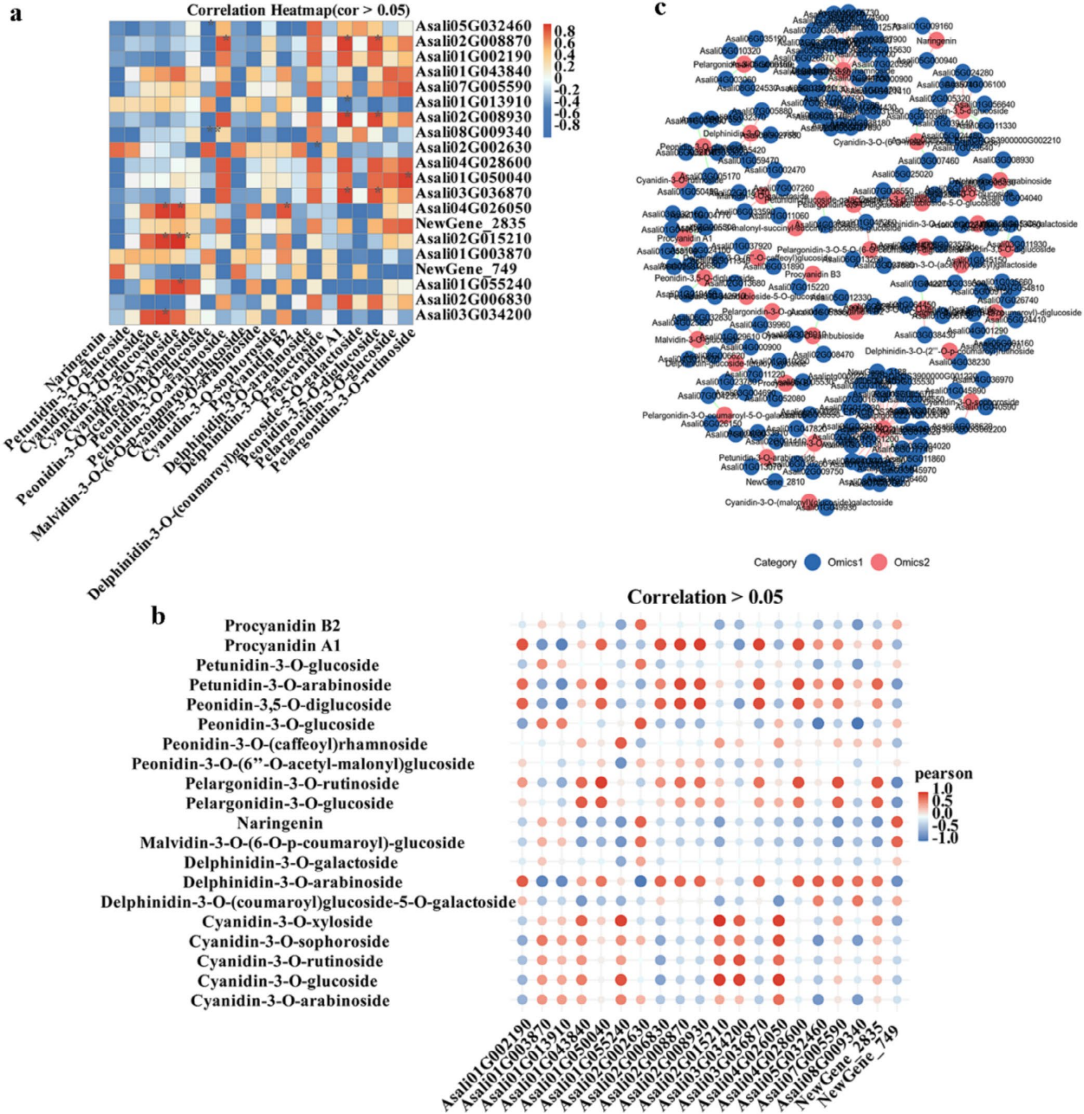
Correlation Heatmap (a), Correlation scatter bubble chart (b), and Gene–Metabolite Co‐expression Regulatory Network (c).

The correlation heatmap (Figure 8a) revealed significant expression‐accumulation associations between 16 candidate DEGs and 18 anthocyanin/flavonoid metabolites. Multiple genes, such as *Asali03G034200*, *Asali04G026050*, *Asali01G050040*, and so on, exhibited strong positive correlations with core anthocyanin glycosides, including Cyanidin‐3‐O‐rutinoside, Cyanidin‐3‐O‐glucoside, Pelargonidin‐3‐O‐rutinoside, and Pelargonidin‐3‐O‐glucoside, with the highest correlation coefficient reaching 0.8. Moreover, these three genes all belonged to the modules showing highly significant correlations with anthocyanin content, namely purple, lightpink3, cyan, antiquewhite2, and darkgreen. This indicates that these genes may act as key positive regulators of the anthocyanin biosynthesis pathway. Meanwhile, several genes showed significant negative correlations with these metabolites, suggesting their potential roles in the negative regulation of anthocyanin synthesis. The correlation bubble plot (Figure 8b) further validated these findings, visually distinguishing the significance of correlations via bubble size and identifying multiple pairs of DEGs and metabolites with high correlation and statistical significance, which provided reliable candidate targets for subsequent regulatory network construction.

Based on the correlation analysis results, we constructed a gene‐metabolite co‐expression regulatory network (Figure 8c). In this network, blue nodes represent differentially expressed genes (DEGs, Omics1), red nodes denote anthocyanin‐related metabolites (Omics2), and edges indicate significant correlations between nodes (|cor| > 0.8, *p* < 0.05). The results demonstrated that multiple DEGs formed tightly co‐regulated modules with various anthocyanin metabolites. Among these DEGs, several served as hub genes in the network, exhibiting significant associations with more than ten anthocyanin metabolites and thus were identified as core candidate genes regulating fruit coloration. Furthermore, the network clearly illustrated the corresponding regulatory gene sets for different anthocyanin metabolites, thereby clarifying the regulatory relationship between gene expression and metabolite accumulation.

In conclusion, the integrated multi‐omics analysis demonstrated that bagging treatments affect the accumulation of core metabolites in the anthocyanin biosynthesis pathway by regulating the expression of key DEGs, ultimately leading to differences in fruit color. The core regulatory genes and co‐expression modules screened in this study provide important omics‐based evidence for further elucidating the molecular mechanism of anthocyanin synthesis in *Zuili* fruits and optimizing bagging cultivation techniques.

### 3.6 RT‐qPCR Validation of Differentially Expressed Genes

**FIGURE 9:**
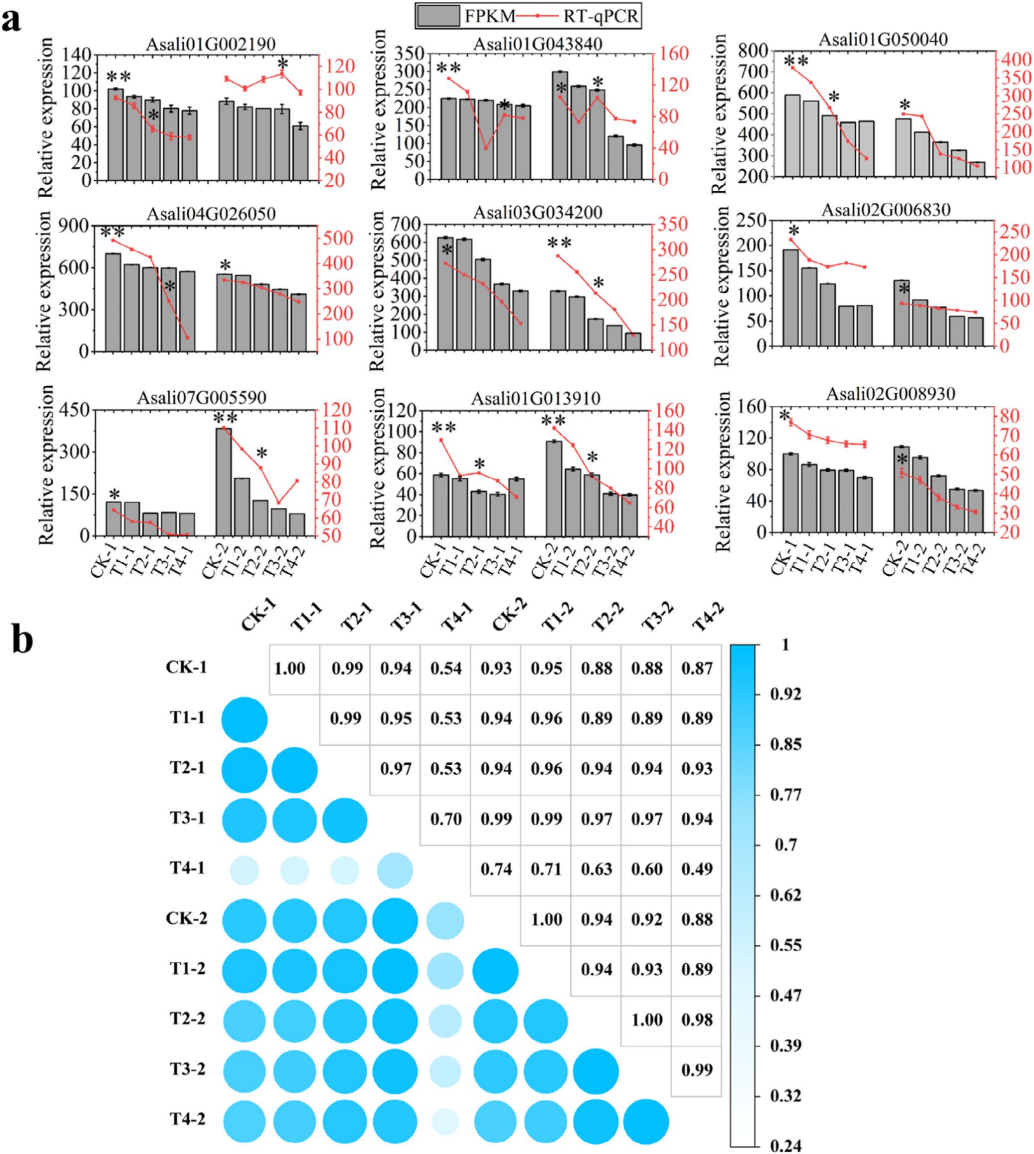
Figure validation of transcriptome data reliability and correlation analysis between candidate genes and anthocyanin biosynthesis‐related genes. (a) Expression profiles of 9 selected differentially expressed genes (DEGs) validated by RT‐qPCR. Gray bars represent FPKM values from RNA‐seq, and red lines represent relative expression levels from RT‐qPCR. Data are presented as means ± standard error (*n* = 3). Asterisks indicate significant differences (*p < 0.05, **p < 0.01). (b) Pearson correlation analysis between the expression levels of three high‐expression candidate genes (Asali03G034200, Asali04G026050, Asali01G050040) and known anthocyanin biosynthesis‐related genes across all samples. The color gradient indicates the correlation coefficient (*r*), ranging from 0.24 (light blue) to 1.00 (dark blue).

RT‐qPCR validation confirmed that the expression trends of 9 randomly selected DEGs were consistent with the RNA‐seq data, verifying the reliability of the transcriptome dataset (Figure 9). Pearson correlation analysis further revealed that three high‐expression candidate genes (*Asali03G034200*, *Asali04G026050*, and *Asali01G050040*) were significantly positively correlated with key genes in the anthocyanin biosynthesis pathway (*r* > 0.90, *p* < 0.01), indicating their potential roles in anthocyanin synthesis and regulation. Detailed annotations of candidate DEGs are provided in Table S4.

## 4 Discussion

Anthocyanins, as key flavonoid pigments that determine the appearance and nutritional value of *P. salicina* fruit, are regulated by light signals during their synthesis and accumulation (Wang et al. 2026; Zhang et al. 2026).

In this study, *Zuili* was used as experimental material, and bagging treatments with different light transmittance rates were set up. Combined with transcriptomic and metabolomic analyses, the regulatory mechanism of light on anthocyanin biosynthesis was systematically investigated. The results showed that anthocyanin accumulation in the pericarp of *Zuili* was significantly positively correlated with fruit bag light transmittance. The total anthocyanin content in the high‐light‐transmission groups (CK, T1) was significantly higher than that in the low‐light‐transmission groups (T2–T4), confirming that light is an indispensable environmental factor for anthocyanin biosynthesis in *Zuili*. This consistent regulatory pattern has also been documented in perennial fruit tree species, including apple (Bu et al. 2025), sweet cherry (Colletti et al. 2025), and pear (Sun et al. 2025). Implying a conserved light‐dependent regulatory mode of anthocyanin synthesis.

In the anthocyanin biosynthetic pathway, the upstream genes of the phenylpropanoid pathway, including *PAL*, *C4H*, and *4CL*, showed an upregulated trend under low‐light conditions (T3, T4), implying that low light may induce the supply of downstream precursor substances (Zhu et al. 2017). Notably, the late biosynthetic gene ANS was significantly upregulated under both low‐light and dark bagging conditions in this study. We further clarified that the shaded microenvironment created by low‐light and dark bagging induced elevated ANS transcription, which may represent a stress adaptive response of *Zuili* fruit to weak‐light stress rather than a direct signal driving metabolite accumulation. Here, we precisely described the upregulation trend of ANS under reduced light without overinterpreting its physiological consequence, and rationally explained its expression discrepancy across different light transmittance treatments.

By contrast, the key structural genes in the flavonoid branch, especially *CHS*, were highly expressed under high‐light conditions, indicating that sufficient light is a prerequisite for activating rate‐limiting steps and effectively directing metabolic flux toward anthocyanin biosynthesis (Yang et al. 2021). Intriguingly, although *ANS* transcription was induced under dark conditions, almost no anthocyanin accumulation was detected by metabolomic analysis in the dark group, forming an obvious transcription–metabolite inconsistency. This decoupling indicates that transcriptional upregulation alone cannot drive anthocyanin biosynthesis under light‐deficient conditions, implying that multiple post‐transcriptional and post‐translational layers dominate the final metabolic phenotype.

Beyond transcriptional regulation, light may govern anthocyanin accumulation through multiple sophisticated regulatory layers. Post‐transcriptional regulation, including alternative splicing, microRNA‐mediated silencing and RNA stability modulation, can constrain the translation efficiency of anthocyanin structural genes even when transcript abundance is elevated (Zhu et al. 2023). Furthermore, light‐dependent enzyme activity provides an essential post‐translational constraint: the catalytic activity of *ANS* and other key biosynthetic enzymes is strictly light‐dependent, and high transcript levels cannot guarantee functional enzyme activation under dark or extremely low‐light conditions (Zhang et al. 2025). In addition, anthocyanin transport mechanisms mediated by *GST* and *MATE* family transporters are indispensable for sequestering synthesized anthocyanins into vacuoles; insufficient expression or functional suppression of these transporters under weak light may block metabolite storage, even if upstream synthetic genes are transcriptionally activated (Li et al. 2026). Moreover, anthocyanin degradation pathways are also light‐modulated: low‐light or dark environments may activate degradation‐related enzymes, accelerating the catabolism of newly synthesized anthocyanins and offsetting the transcriptional induction of biosynthetic genes (Piryaei and Khiavi 2026). Collectively, light not only participates in the transcriptional activation of biosynthetic genes but also acts as a crucial regulator of post‐transcriptional processing, enzyme functional activation, transporter‐mediated trafficking, and degradation pathway repression (Ramzan et al. 2026). Therefore, the expression level of late biosynthetic genes cannot fully represent the actual accumulation of metabolites, highlighting the necessity of integrated transcriptomic and metabolomic validation in deciphering light‐regulated anthocyanin formation.

WGCNA combined with transcriptome‐metabolome correlation analysis revealed that 9 differentially expressed genes exhibited significant expression–accumulation associations with 18 anthocyanin and flavonoid metabolites. Among them, genes including *Asali03G034200*, *Asali04G026050*, and *Asali01G050040* were highly positively correlated with core anthocyanin glycosides such as cyanidin‐3‐O‐rutinoside, cyanidin‐3‐O‐glucoside, pelargonidin‐3‐O‐rutinoside, and pelargonidin‐3‐O‐glucoside, with some correlation coefficients reaching 0.8. Further correlation analysis also proved that these three candidates had strong positive correlations with well‐characterized anthocyanin biosynthetic structural genes, further supporting their close linkage to the anthocyanin pathway. Gene‐metabolite co‐expression regulatory networks revealed that multiple differentially expressed genes formed tight co‐regulated modules with anthocyanin metabolites (Zong et al. 2025). Several hub genes were significantly associated with more than ten metabolites simultaneously, and were thus regarded as key candidate genes regulating fruit coloration. Accumulative multi‐omics studies on stone fruits and other horticultural crops have demonstrated that such highly expressed hub genes, despite not being classic catalytic structural genes, act as essential co‐regulators. They work together with conventional synthetic genes to mediate light‐induced color formation, metabolite transport and stress responses related to pigment accumulation (Xiong et al. 2025; Zhu et al. 2025). The specific functions of these candidate genes, their upstream and downstream transcriptional regulatory networks, and their actual genetic effects on anthocyanin biosynthesis still require in‐depth validation via molecular experiments, including transgenic functional complementation, yeast one‐hybrid assay, and dual‐luciferase reporter system.

In the integrated multi‐omics analysis, the expression of key structural genes showed consistent trends with the accumulation of characteristic anthocyanin metabolites such as cyanidin‐3‐O‐glucoside and pelargonidin‐3‐O‐glucoside, supporting the conserved regulatory cascade of “light signal‐gene expression‐metabolite accumulation” (Nidhi et al. 2024). Nevertheless, current omics evidence only reflects correlative links and cannot construct a complete causal regulatory chain. Moderate correlation coefficients between transcriptomic profiles and metabolite abundance further suggest that anthocyanin accumulation is controlled by multi‐layered regulatory networks, covering post‐transcriptional processing, protein modification, transporter‐mediated intercellular trafficking, and active degradation, rather than being simply determined at the transcriptional level alone.

From the perspective of practical cultivation, the T1 treatment (50% light transmittance) not only maintained a high level of anthocyanin accumulation but also improved fruit appearance and pericarp smoothness, providing a theoretical basis for optimizing bagging strategies in *Zuili* production. This study also implies that delaying bagging time or implementing timely pre‐harvest bag removal to increase light exposure can effectively promote fruit coloration. Importantly, stable anthocyanin accumulation cannot be sustained under persistent low‐light or dark environments, even with obvious transcriptional upregulation of late structural genes such as *ANS*.

In summary, this study demonstrates that light transmittance significantly modulates anthocyanin biosynthesis in *Zuili* fruit by reprogramming genome‐wide gene expression profiles. The transcriptional induction of late anthocyanin biosynthetic genes under dark and shaded conditions fails to drive effective anthocyanin accumulation, uncovering a remarkable decoupling between transcriptomic alteration and metabolic phenotype. Multi‐omics association analyses in this work only support correlative relationships rather than deterministic causal regulatory mechanisms, and the biological functions of identified hub genes remain to be verified via further molecular functional experiments. Collectively, these findings provide valuable candidate gene resources and a theoretical foundation for precision bagging cultivation optimization in *Zuili*, while the elaborate molecular mechanism underlying light‐modulated anthocyanin biosynthesis still warrants deeper mechanistic exploration in future research.

## 5 Conclusion

Using *Zuili* as material, this study conducted bagging treatments with different light transmittances and a control, and systematically explored the regulatory effect of light on anthocyanin biosynthesis via transcriptomic and metabolomic analyses. The results showed that anthocyanin content increased significantly with higher light transmittance, dominated by cyanidin and pelargonidin derivatives. High light markedly induced the expression of key structural genes such as *CHS*, whereas low light upregulated some related genes but failed to promote anthocyanin accumulation. Multi‐omics analysis established the linkage of “light transmittance–gene–metabolite” and identified genes with high absolute expression levels (FPKM > 300), including *Asali03G034200*, *Asali04G026050*, *Asali01G050040*, and so on. In practice, 50% light transmittance bags balanced fruit coloration and smoothness, and delayed bagging or pre‐harvest bag removal improved coloration. This study provides gene resources and a theoretical basis for color improvement and precise bagging of *Zuili* plum, and the light‐regulated molecular network can be further refined in future research.

## Author Contributions

**Yang Liu:** data curation. **Zhongxing Zhang:** investigation. **Qihua Lu:** supervision. **Jun Li:** methodology. **Jiangong Wang:** formal analysis. **Yanlong Gao:** software. **Yanbiao Li:** software, writing – review and editing, resources.

## Funding

Jiaxing City Public Welfare Research Project (2024AY10058); Zhejiang Provincial Natural Science Foundation Project (LJXSQY26C200002); Jiaxing Municipal Science and Technology Project Public Welfare Research Special Fund (2025CGZ056).

## Conflicts of Interest

The authors declare no conflicts of interest.

## Supporting information


**Figure S1:** Functional classification of consensus sequences. (a) COG functional classification of consensus sequences. The x‐axis represents the COG functional categories, and the y‐axis represents the frequency of genes in each category. (b) GO functional annotation of consensus sequences. Genes are classified into three main categories: cellular component (blue), molecular function (red), and biological process (green). The left y‐axis shows the percentage of genes, and the right y‐axis shows the corresponding number of genes in each GO term.
**FIGURE S2:** Soft Threshold Screening and Scale‐Free Network Construction in WGCNA. Scale Independence and Mean Connectivity (a), Histogram of Connectivity with Power and Check Scale Free Topology (b).

**Table S1:** Primer information used in this study.
**Table S2:** Anthocyanin Monomers.
**Table S3:** Summary of transcriptome sequencing data.
**Table S4:** Annotation information of candidate genes.

## Data Availability

The data that support the findings of this study are available on request from the corresponding author. The data are not publicly available due to privacy or ethical restrictions.
