# Supplementary material for: Elucidating the Molecular Mechanisms Underlying Fruit Bag‐Mediated Light Transmittance and Its Effects on Anthocyanin Biosynthesis in Chinese Plum (Prunus salicina ‘Longzhong’): An Integrated Transcriptomic and Metabolomic Analysis
Source: Food Sci Nutr. 2026 Jul 15;14(7):e72077. doi: 10.1002/fsn3.72077 (PMC13373313; doi:10.1002/fsn3.72077)
Supplement: Supplementary file 1 — Figure S1: Functional classification of consensus sequences. (a) COG functional classification of consensus sequences. The x‐axis represents the COG functional categories, and the y‐axis represents the frequency of genes in each category. (b) GO functional annotation of consensus sequences. Genes are classified into three main categories: cellular component (blue), molecular function (red), and biological process (green). The left y‐axis shows the percentage of genes, and the right y‐axis shows the corresponding number of genes in each GO term. FIGURE S2: Soft Threshold Screening and Scale‐Free Network Construction in WGCNA. Scale Independence and Mean Connectivity (a), Histogram of Connectivity with Power and Check Scale Free Topology (b). [file FSN3-14-e72077-s001.docx]

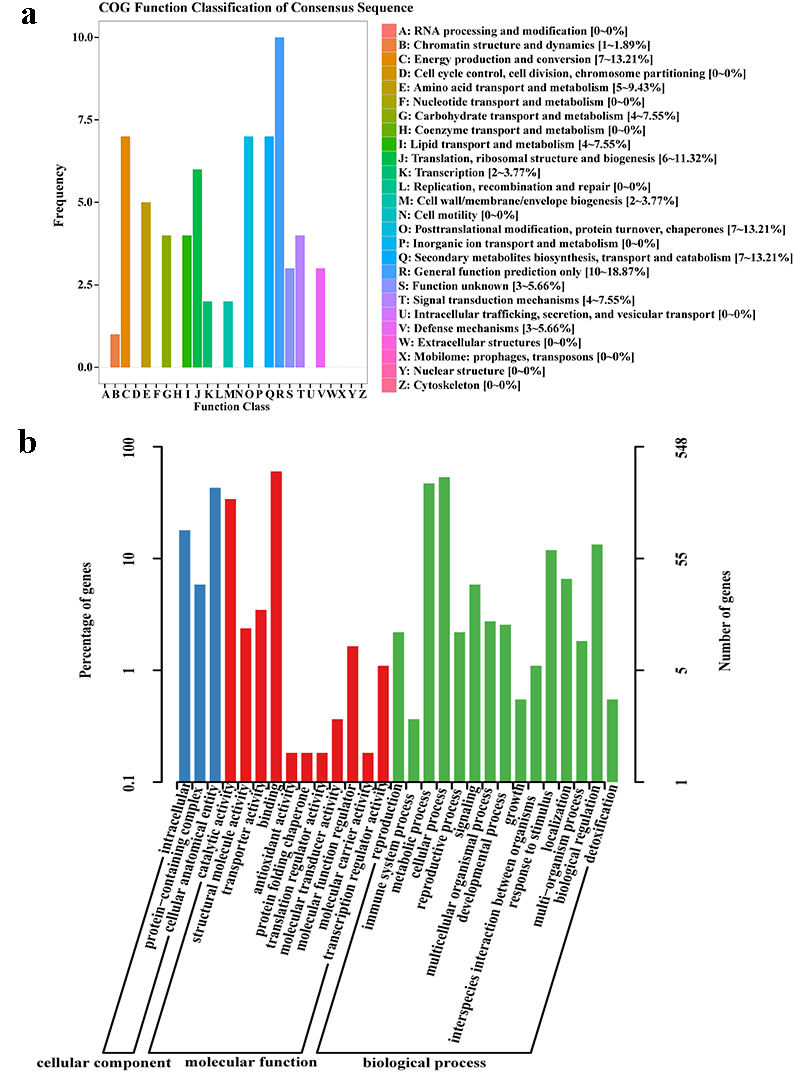


FIGURE S1 Functional classification of consensus sequences. (a) COG functional classification of consensus sequences. The x-axis represents the COG functional categories, and the y-axis represents the frequency of genes in each category. (b) GO functional annotation of consensus sequences. Genes are classified into three main categories: cellular component (blue), molecular function (red), and biological process (green). The left y-axis shows the percentage of genes, and the right y-axis shows the corresponding number of genes in each GO term.


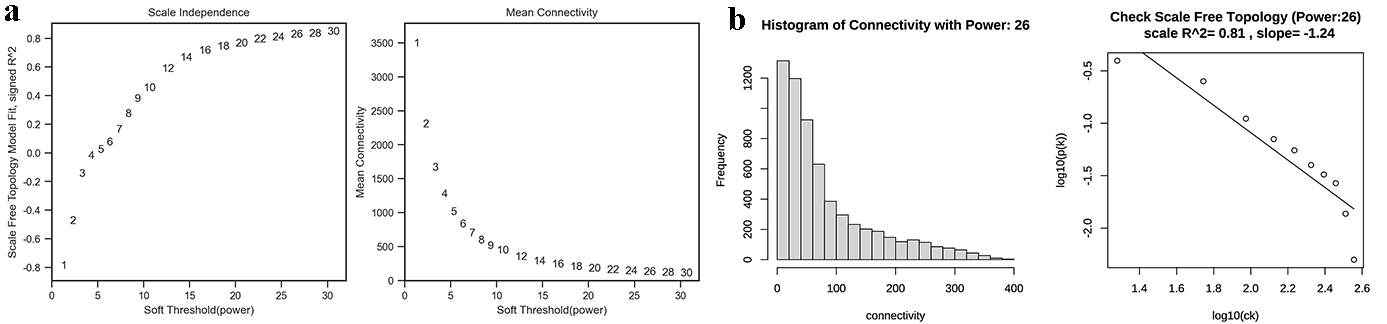


FIGURE S2 Soft Threshold Screening and Scale-Free Network Construction in WGCNA. Scale Independence and Mean Connectivity (a), Histogram of Connectivity with Power and Check Scale Free Topology (b)
