# Supplementary material for: Elucidating the Molecular Mechanisms Underlying Fruit Bag‐Mediated Light Transmittance and Its Effects on Anthocyanin Biosynthesis in Chinese Plum (Prunus salicina ‘Longzhong’): An Integrated Transcriptomic and Metabolomic Analysis
Source: Food Sci Nutr. 2026 Jul 15;14(7):e72077. doi: 10.1002/fsn3.72077 (PMC13373313; doi:10.1002/fsn3.72077)
Supplement: Supplementary file 2 — Table S1: Primer information used in this study. Table S2: Anthocyanin Monomers. Table S3: Summary of transcriptome sequencing data. Table S4: Annotation information of candidate genes. [file FSN3-14-e72077-s002.docx]

Table S1 Primer information used in this study

| **Gene ID** | **Primer sequence** |
| --- | --- |
| *Asali05G032460* | F:GCGCTGATTACTCCGATGGA; R:CATGCAGGGCAAAAACACCA |
| *Asali02G008870* | F:AGCAACGTCTTCGATCCGTT; R:GGCTTCTGGGGTCTCTTTCC |
| *Asali01G002190* | F:ACGTTGGATTCGTGTGTGGT; R:GGCGGTTAAAGGGAGTCCAA |
| *Asali01G043840* | F:CCACCATGTCTGCTTCGAGT; R:CCTACTACTGCAGGCTCAGC |
| *Asali07G005590* | F:CACAGAGCAGCTACACCACA; R:GTCACTCATGCCCTGCTTCT |
| *Asali01G013910* | F:CTACAAGGAGGGTGCCACTG; R:TGAAGCCAGCTGTGCCAATA |
| *Asali02G008930* | F:TGGGGGATGCCAATTCAGTC; R:ACCTCATTCACCTTCGCGTT |
| *Asali08G009340* | F:CCCATTGATGATTGCTGGCG; R:ATGTCGTAGAGTCCCTGGCT |
| *Asali02G002630* | F:GTGCTTCTGGTGATGGAGCT; R:ATGGACATTGTGGAGCTGCA |
| *Asali04G028600* | F:CATGCAGGGCAAAAACACCA; R:GGGACAAGGAGGGTAGTTGC |
| *Asali01G050040* | F:CGTCACCGTCCTTTCTGTCA; R:AAGCAGAGATGAACGACGGG |
| *Asali03G036870* | F:TCACCGACGTCTTCACTGTG; R:AAAGTAACAACACACGCGCC |
| *Asali04G026050* | F:AAGCAGAGATGAACGACGGG; R:CACAACAGCAACACAAGGCA |
| *Asali02G015210* | F:AAAGTAACAACACACGCGCC; R:TGGGGGATGCCAATTCAGTC |
| *Asali01G055240* | F:ACCTCCGCCTCTTGTTTCTG; R:CCACCATGTCTGCTTCGAGT |
| *Asali02G006830* | F:GGCTTCTGGGGTCTCTTTCC; R:GTGCTTCTGGTGATGGAGCT |
| *Asali03G034200* | F:GGCGGTTAAAGGGAGTCCAA; R:GTGAGGGGATGCAAACCTGA |
| *Actin* | F:GATGCTGAGGATATTCAACCCC;R:CCATGACACCAGTATGACGAGG |

Table S2 Anthocyanin Monomers

| **Compounds** | **Class** | **Molecular Weight** | **Formula** |
| --- | --- | --- | --- |
| Quercetin-3-O-glucoside | Flavonoid | 464.0954761 | C_21_H_20_O_12_ |
| Delphinidin-3-O-(6-O-p-coumaroyl)-glucoside | Delphinidin | 611.1400805 | C_30_H_27_O_14_ |
| Peonidin-3-O-xyloside | Peonidin | 433.1134719 | C_21_H_21_O_10_ |
| Cyanidin-3-O-rutinoside | Cyanidin | 595.1662953 | C_27_H_31_O_15_ |
| Procyanidin B3 | Procyanidin | 578.1424263 | C_30_H_26_O_12_ |
| Procyanidin B2 | Procyanidin | 578.1424263 | C_30_H_26_O_12_ |
| Malvidin-3-O-(6-O-p-coumaroyl)-glucoside | Malvidin | 639.1713807 | C_32_H_31_O_14_ |
| Procyanidin B1 | Procyanidin | 578.1424263 | C_30_H_26_O_12_ |
| Pelargonidin-3-O-coumaroyl-5-O-galactoside | Pelargonidin | 579.1502513 | C_30_H_27_O_12_ |
| Cyanidin-3-O-xyloside | Cyanidin | 419.0978218 | C_20_H_19_O_10_ |
| Procyanidin A1 | Procyanidin | 576.1267762 | C_30_H_24_O_12_ |
| Cyanidin-3-xylosyl-galactoside | Cyanidin | 581.1506452 | C_26_H_29_O_15_ |
| Peonidin-3-O-sambubioside-5-O-glucoside | Peonidin | 757.2191187 | C_33_H_41_O_20_ |
| Petunidin-3-O-arabinoside | Petunidin | 449.1083865 | C_21_H_21_O_11_ |
| Delphinidin-3-O-galactoside | Delphinidin | 465.1033011 | C_21_H_21_O_12_ |
| Peonidin-3-O-(caffeoyl)rhamnoside | Peonidin | 609.160816 | C_31_H_29_O_13_ |
| Cyanidin-3-O-(malonyl)(glucoside)galactoside | Cyanidin | 697.1616038 | C_30_H_33_O_19_ |
| Delphinidin-3-O-arabinoside | Delphinidin | 435.0927364 | C_20_H_19_O_11_ |
| Delphinidin-3-O-rutinoside | Delphinidin | 611.1612099 | C_27_H_31_O_16_ |
| Delphinidin-3-O-sambubioside-5-O-glucoside | Delphinidin | 759.1983833 | C_32_H_39_O_21_ |
| Cyanidin-3-O-sophoroside | Cyanidin | 611.1612099 | C_27_H_31_O_16_ |
| Cyanidin-3-O-arabinosidase-glucoside | Cyanidin | 581.1506452 | C_26_H_29_O_15_ |
| Cyanidin-3-O-arabinoside | Cyanidin | 419.0978218 | C_20_H_19_O_10_ |
| Cyanidin-3-O-glucoside | Cyanidin | 449.1083865 | C_21_H_21_O_11_ |
| Peonidin-3-O-glucoside | Peonidin | 463.1240366 | C_22_H_23_O_11_ |
| Peonidin-3-O-(6''-O-acetyl-malonyl)glucoside | Peonidin | 591.1349952 | C_27_H_27_O_15_ |
| Cyanidin-3-O-(6''-O-coumaryl)xyloside | Cyanidin | 565.1346012 | C_29_H_25_O_12_ |
| Cyanidin-3-pentoside | Cyanidin | 419.09727 | C_20_H_19_O_10_ |
| Malvidin-3-O-galactoside | Malvidin | 493.1346012 | C_23_H_25_O_12_ |
| Delphinidin-3-O-glucoside | Delphinidin | 465.1033011 | C_21_H_21_O_12_ |
| Naringenin | Flavonoid | 272.0684735 | C_15_H_12_O_5_ |
| Cyanidin-3-O-(6-O-malonyl-beta-D-glucoside) | Cyanidin | 535.1087804 | C_24_H_23_O_14_ |
| Peonidin-3-O-sophoroside | Peonidin | 625.17686 | C_28_H_33_O_16_ |
| Peonidin-3,5-diglucoside | Peonidin | 625.17686 | C_28_H_33_O_16_ |
| Peonidin-3,5-O-diglucoside | Peonidin | 625.17686 | C_28_H_33_O_16_ |
| Pelargonidin-3-O-rutinoside | Pelargonidin | 579.1713807 | C_27_H_31_O_14_ |
| Peonidin-3-O-(6''-O-acetyl)glucoside | Peonidin | 505.1346012 | _C24H25O12_ |
| Pelargonidin-3-O-sophoroside | Pelargonidin | 595.1662953 | C_27_H_31_O_15_ |
| Pelargonidin-3-O-glucoside | Pelargonidin | 433.1134719 | C_21_H_21_O_10_ |
| Cyanidin-3-O-sambubioside | Cyanidin | 581.1506452 | C_26_H_29_O_15_ |
| Cyanidin-3-(6''-O-p-caffeoyl)-glucoside | Cyanidin | 611.1400805 | C_30_H_27_O_14_ |
| Delphinidin-3-O-rhamnoside | Delphinidin | 449.10839 | C_21_H_21_O_11_ |
| Petunidin-3-O-glucoside | Petunidin | 479.1189512 | C_22_H_23_O_12_ |
| Peonidin-3-O-galactoside | Peonidin | 463.1240366 | C_22_H_23_O_11_ |
| Malvidin-3-O-glucoside | Malvidin | 493.1346012 | C_23_H_25_O_12_ |
| Pelargonidin-3-O-5-O-(6-O-coumaroyl)-diglucoside | Pelargonidin | 741.2030747 | C_36_H_37_O_17_ |
| Malvidin-3-O-(6''-O-feruloyl)galactoside | Malvidin | 669.1819454 | C_33_H_33_O_15_ |
| Malvidin-3-O-galactoside | Malvidin | 493.1346012 | C_23_H_25_O_12_ |
| Cyanidin-3-O-(6''-O-coumaryl)galactoside | Cyanidin | 595.1451659 | C_30_H_27_O_13_ |
| Cyanidin-3-O-(6''-O-acetyl-2''-O-xylosyl)glucoside | Cyanidin | 623.1612099 | C_28_H_31_O_16_ |
| Delphinidin-3,5-O-diglucoside | Delphinidin | 627.1561245 | C_27_H_31_O_17_ |
| Malvidin-3-O-arabinoside | Malvidin | 463.1240366 | C_22_H_23_O_11_ |
| Delphinidin-3-O-(coumaroyl)glucoside-5-O-galactoside | Delphinidin | 773.192904 | C_36_H_37_O_19_ |
| Cyanidin-3-O-(6''-O-caffeoyl)rhamnoside | Cyanidin | 595.1451659 | C_30_H_27_O_13_ |
| Cyanidin-3-malonyl-succinyl-succinyl-glucoside-glucoside | Cyanidin | 897.1936918 | C_38_H_41_O_25_ |
| Cyanidin-3-O-rutinoside-glucoside | Cyanidin | 757.2191187 | C_33_H_41_O_20_ |
| Cyanidin-3-O-(6''-O-ferulyl-xylosyl)glucoside | Cyanidin | 757.1979893 | C_36_H_37_O_18_ |
| Petunidin-glucoside-galactoside | Petunidin | 641.1717746 | C_28_H_33_O_17_ |
| Pelargonidin-3-O-(6-O-malonyl-beta-D-glucoside) | Pelargonidin | 519.1138658 | C_24_H_23_O_13_ |
| Delphinidin-3-O-rutinoside-5-O-glucoside | Delphinidin | 773.2140333 | C_33_H_41_O_21_ |
| Malvidin-3-O-xyloside | Malvidin | 463.1240366 | C_22_H_23_O_11_ |
| Delphinidin-glucoside-feruloyl-xyloside | Delphinidin | 773.2140333 | C_33_H_41_O_21_ |
| Cyanidin-3-O-5-O-(6-O-coumaroyl)-diglucoside | Cyanidin | 757.1979893 | C_36_H_37_O_18_ |
| Cyanidin-3-O-(acetyl)(xylosyl)galactoside | Cyanidin | 623.1612099 | C_28_H_31_O_16_ |
| Pelargonidin-3,5-O-diglucoside | Pelargonidin | 595.1662953 | C_27_H_31_O_15_ |
| Delphinidin-3-O-sophoroside | Delphinidin | 627.1561245 | C_27_H_31_O_17_ |
| Cyanidin-3-O-sambubioside-5-O-glucoside | Cyanidin | 743.2034686 | C_32_H_39_O_20_ |
| Delphinidin-3-O-(6''-O-caffeoyl)glucoside | Delphinidin | 627.1349952 | C_30_H_27_O_15_ |
| Malvidin-3,5-O-diglucoside | Malvidin | 655.1874246 | C_29_H_35_O_17_ |

Table S3 Summary of transcriptome sequencing data

| SampleID | ReadSum | BaseSum | GC(%) | Q20(%) | Q30(%) |
| --- | --- | --- | --- | --- | --- |
| CK-1-1 | 19160022 | 5718431402 | 45.27 | 99.91 | 99.04 |
| CK-1-2 | 19951773 | 5959112205 | 45.77 | 99.79 | 98.51 |
| CK-1-3 | 23528158 | 7016871881 | 45.82 | 99.91 | 99.04 |
| CK-2-1 | 22654915 | 6765851710 | 45.56 | 99.65 | 97.96 |
| CK-2-2 | 20094074 | 6001118499 | 45.21 | 99.77 | 98.43 |
| CK-2-3 | 21143222 | 6310069509 | 45.25 | 99.69 | 98.14 |
| T1-1-1 | 20548385 | 6135874225 | 46.05 | 99.82 | 98.66 |
| T1-1-2 | 20710304 | 6184478260 | 45.42 | 99.72 | 98.28 |
| T1-1-3 | 19995854 | 5978319157 | 45.36 | 99.72 | 98.18 |
| T1-2-1 | 20571992 | 6149439030 | 45.56 | 99.75 | 98.47 |
| T1-2-2 | 20497495 | 6126113391 | 45.58 | 99.75 | 98.41 |
| T1-2-3 | 22828963 | 6824886535 | 45.45 | 99.56 | 97.55 |
| T2-1-1 | 20864840 | 6235855057 | 45.38 | 99.69 | 98.08 |
| T2-1-2 | 20132178 | 6016158516 | 45.23 | 99.82 | 98.68 |
| T2-1-3 | 19896782 | 5935353899 | 45.33 | 99.92 | 99.17 |
| T2-2-1 | 21283395 | 6359674379 | 45.35 | 99.64 | 97.84 |
| T2-2-2 | 19969472 | 5973512699 | 45.24 | 99.69 | 98.06 |
| T2-2-3 | 22531122 | 6735373129 | 45.3 | 99.47 | 97.07 |
| T3-1-1 | 20281104 | 6060720425 | 45.13 | 99.78 | 98.4 |
| T3-1-2 | 20637500 | 6166967809 | 45.33 | 99.72 | 98.22 |
| T3-1-3 | 20901585 | 6247633926 | 45.22 | 99.73 | 98.24 |
| T3-2-1 | 19870252 | 5936083561 | 45.01 | 99.72 | 98.22 |
| T3-2-2 | 20593746 | 6156714297 | 45.1 | 99.69 | 98.06 |
| T3-2-3 | 21069159 | 6297209974 | 45.1 | 99.67 | 98.08 |
| T4-1-1 | 21335787 | 6375836900 | 45 | 99.65 | 97.95 |
| T4-1-2 | 20688287 | 6184944957 | 44.9 | 99.69 | 98.09 |
| T4-1-3 | 22223600 | 6643064140 | 45.19 | 99.61 | 97.72 |
| T4-2-1 | 20324818 | 6074913211 | 45.38 | 99.74 | 98.28 |
| T4-2-2 | 20404061 | 6095336513 | 45.33 | 99.66 | 97.99 |
| T4-2-3 | 20337226 | 6080988636 | 45.34 | 99.71 | 98.14 |

Table S4 Annotation information of candidate genes

| **Gene ID** | **Annotation** |
| --- | --- |
| *Asali05G032460* | abscisic stress-ripening protein 5-like |
| *Asali02G008870* | CI small heat shock protein 2 |
| *Asali01G002190* | unnamed protein product |
| *Asali01G043840* | dehydrin COR47-like |
| *Asali07G005590* | protein E6-like |
| *Asali01G013910* | translationally-controlled tumor protein homolog |
| *Asali02G008930* | unnamed protein product |
| *Asali08G009340* | pectase lyase |
| *Asali02G002630* | acidic endochitinase |
| *Asali04G028600* | 1-amino-cyclopropane-1-carboxylic acid oxidase |
| *Asali01G050040* | hypothetical protein PRUPE |
| *Asali03G036870* | Thiamine pyrophosphate enzyme |
| *Asali04G026050* | unnamed protein product |
| *Asali02G015210* | BAG family molecular chaperone regulator 6 |
| *Asali01G055240* | probable E3 ubiquitin-protein ligase RZFP34 |
| *Asali02G006830* | superoxide dismutase |
| *Asali03G034200* | metallothionein-like protein |
